# Comparative Remineralization Efficacy of Different Physical Forms of Chitosan in a Newly Developed Olive Leaf Extract-Containing Artificial Saliva

**DOI:** 10.3390/molecules31203601

**Published:** 2026-10-10

**Authors:** Aslı Silkü Oflioğlu, Deniz Çetin, Hülya Erten Can, Pınar Güvenç, İdil Tekin, Hanife Altınışık, Shakhrukh Abdukadirov

**Affiliations:** 1 Department of Restorative Dentistry, Faculty of Dentistry, Dokuz Eylül University, Izmir 35340, Türkiye; 2 Science and Technology Application and Research Center, Dokuz Eylül University, Izmir 35390, Türkiye; 3 Department of Restorative Dentistry, Faculty of Dentistry, Gazi University, Ankara 06490, Türkiye; 4 Faculty of Dentistry, Khoja Akhmet Yassawi International Kazakh-Turkish University, Turkistan 161200, Kazakhstan

**Keywords:** chitosan, enamel remineralization, olive leaf extract, artificial saliva, tooth bleaching, natural biopolymer

## Abstract

Plant-derived bioactive compounds and natural biopolymers are increasingly investigated as components of multifunctional remineralization systems. This in vitro study compared the remineralization efficacy of chitosan film, gel, and solution on bleached human enamel within an olive leaf extract-containing artificial saliva system. The novelty of the present study lies in the direct comparison of three physical presentations of chitosan—film, gel, and solution—within a common olive leaf extract-containing artificial saliva platform. Olive leaf extract was incorporated as a common component of the artificial saliva in all groups and was not evaluated as an independent experimental factor. Seventy-six sound human anterior teeth were used; 60 were randomly allocated to four groups (*n* = 15) for Vickers microhardness analysis, and 16 (*n* = 4/group) were evaluated by SEM–EDX. Following bleaching with 35% hydrogen peroxide, specimens underwent 28-day remineralization protocols. Microhardness was measured at baseline, after bleaching, and after remineralization. Bleaching significantly reduced microhardness, whereas remineralization significantly increased it in all groups (*p* < 0.001). Microhardness gain (ΔVHN) differed among groups (*p* < 0.001), with all chitosan formulations showing greater gains than the control (*p* < 0.05). The solution showed the greatest numerical ΔVHN and a significantly greater gain than the gel (*p* = 0.015), but not the film. EDX revealed significant between-group differences in Ca gain (*p* = 0.017) and Ca/P ratio change (*p* = 0.033). Chitosan enhanced post-bleaching enamel microhardness recovery, with solution and film formulations showing particularly favorable remineralization responses.

## 1. Introduction

Dental enamel is continuously subjected to cycles of demineralization and remineralization throughout life. Under physiological conditions, saliva maintains this dynamic equilibrium by supplying calcium and phosphate ions, buffering acidic challenges, and facilitating the natural repair of early mineral loss [1]. However, when the balance shifts toward demineralization, enamel undergoes structural changes characterized by increased porosity, decreased surface microhardness, and enhanced susceptibility to subsequent mineral dissolution. These alterations become particularly relevant following exposure to acidic environments or oxidizing agents used during cosmetic dental procedures.

Among contemporary esthetic treatments, in-office tooth bleaching using high-concentration hydrogen peroxide has become one of the most frequently performed minimally invasive procedures [2]. Although highly effective in improving tooth color, hydrogen peroxide has been reported to induce transient mineral loss, increased enamel permeability, alterations in surface morphology, and reductions in enamel microhardness [2]. Consequently, considerable research has focused on developing bioactive remineralization strategies capable of restoring the physicochemical properties of bleached enamel while preserving the esthetic benefits of bleaching treatment [3].

The development of bioactive materials capable of enhancing enamel remineralization has therefore become an important area of research in preventive and restorative dentistry. Although fluoride-based products remain the cornerstone of enamel remineralization, increasing concern regarding multifunctional bioactive materials has stimulated the search for naturally derived alternatives capable of simultaneously promoting mineral recovery and providing additional biological functions, including antioxidant and antimicrobial activities [4]. In this context, naturally occurring polymers and plant-derived bioactive compounds have emerged as promising candidates for next-generation remineralization strategies [5].

Among natural biopolymers, chitosan has attracted considerable attention because of its favorable physicochemical and biological properties. Chitosan is a cationic polysaccharide obtained through the deacetylation of chitin and is characterized by excellent biocompatibility, biodegradability, bioadhesion, and antimicrobial activity [6]. Owing to its positively charged amino groups, chitosan can interact with negatively charged enamel surfaces, facilitating the retention of mineral ions and promoting nucleation processes involved in hydroxyapatite formation [6]. Previous studies have demonstrated that chitosan-containing formulations may enhance mineral deposition, reduce enamel demineralization, and improve the recovery of surface microhardness in experimentally demineralized enamel [7].

Importantly, the effectiveness of chitosan is not only determined by its chemical composition but may also be influenced by its physical presentation. Different physical forms of biomaterials are expected to influence the residence time of the material on the tooth surface, the release profile of bioactive compounds, and the interaction between the biomaterial and dental tissues [6]. Chitosan films provide prolonged contact and sustained release characteristics, whereas chitosan gels offer enhanced adaptability to irregular surfaces and increased moisture retention [8]. In contrast, chitosan solutions may enable rapid and uniform distribution across the enamel surface. Despite these theoretical advantages, the influence of chitosan physical form on enamel remineralization remains insufficiently understood [8].

In parallel with advances in biomaterial-based remineralization, increasing attention has been directed toward phytochemical-rich natural extracts as multifunctional components of oral biomaterials. Among these, olive leaf extract (*Olea europaea* L.) has emerged as a promising natural source of bioactive phenolic compounds, particularly oleuropein, hydroxytyrosol, verbascoside, and flavonoids [9]. These phytochemicals exhibit potent antioxidant, anti-inflammatory, and antimicrobial activities and have been shown to modulate oxidative stress, protect biological tissues from reactive oxygen species (ROS)-mediated damage, and support tissue homeostasis [10].

Within the oral environment, oxidative stress has been associated with inflammatory processes, alterations in salivary homeostasis, and impaired tissue repair, thereby creating conditions that may compromise oral health [11]. Therefore, phytochemical-enriched formulations may provide biological benefits beyond conventional lubrication or ion delivery by creating a microenvironment that favors mineral recovery while simultaneously protecting dental tissues from oxidative damage. Consequently, olive leaf extract represents an attractive natural additive for the development of next-generation bioactive artificial saliva formulations intended to support enamel remineralization [11].

Recently, the incorporation of plant-derived bioactive compounds into oral care formulations has gained significant interest as a strategy to combine remineralization with broader biological functionality [12]. Artificial saliva systems enriched with phytochemicals may serve as bioactive delivery platforms in addition to providing lubrication and ions. In the present study, olive leaf extract was used as a common phytochemical component of the artificial saliva platform rather than as an independent experimental variable. Accordingly, the primary experimental comparison focused on whether different physical formulations of chitosan modified enamel remineralization within the same olive leaf extract-containing environment.

Although chitosan has previously been investigated as a remineralizing biomaterial and as an additive to bleaching or other dental formulations, the influence of its physical presentation on post-bleaching enamel recovery has not been directly compared within a common remineralization environment. The present study addresses this gap by comparing film, gel, and solution formulations of chitosan within the same olive leaf extract-containing artificial saliva platform using complementary Vickers microhardness and SEM–EDX analyses. Therefore, the aim of the present in vitro study was to compare the remineralization potential of chitosan film, gel, and solution within this standardized artificial saliva system. The null hypothesis was that the physical form of chitosan would not significantly influence the remineralization outcomes.

Therefore, the aim of the present in vitro study was to compare the remineralization potential of chitosan film, gel, and solution incorporated into an olive leaf extract-containing artificial saliva using Vickers microhardness measurements and SEM–EDX analyses. The null hypothesis tested was that the physical form of chitosan would not significantly influence the remineralization outcomes achieved within this bioactive artificial saliva system.

## 2. Results

### 2.1. Vickers Surface Microhardness

Vickers surface microhardness results are presented in Table 1. Baseline microhardness values did not differ significantly among the experimental groups (*p* = 0.627), indicating group comparability before bleaching. Mixed-design repeated-measures ANOVA revealed a significant effect of time (*p* < 0.001) and a significant group × time interaction (*p* < 0.001, partial η^2^ = 0.274), demonstrating that the temporal pattern of microhardness change differed among the formulations. The overall group main effect was not significant (*p* = 0.414). Bleaching significantly reduced surface microhardness in all groups, and post-bleaching values differed among groups (*p* = 0.003). Following 28 days of remineralization, microhardness increased significantly in all groups compared with post-bleaching measurements (*p* < 0.001). At T2, a significant group difference was detected (*p* = 0.005). The chitosan solution group exhibited significantly higher final microhardness than the chitosan gel and control groups (*p* = 0.014 and *p* = 0.018, respectively), whereas no significant difference was observed between solution and film (*p* > 0.05). Final microhardness values were significantly higher than baseline in the chitosan film, gel, and solution groups (all adjusted *p* < 0.05), while baseline and final microhardness did not differ significantly in the control group (*p* = 0.819).

### 2.2. Microhardness Gain During Remineralization

Bleaching reduced Vickers microhardness in all four experimental groups, whereas 28 days of remineralization increased microhardness in every group. Numerically, the magnitude of recovery was greatest in the chitosan solution group, followed by the chitosan film, chitosan gel, and control groups. The absolute microhardness gain during remineralization (ΔVHN = T2 − T1) differed significantly among groups (Welch’s ANOVA, *p* < 0.001; Table 1). All chitosan-containing groups demonstrated significantly greater gains than the control group (*p* < 0.05). The chitosan solution group also showed a significantly greater increase than the gel group (*p* = 0.015), whereas solution versus film and film versus gel were not significantly different (*p* > 0.05). Percentage microhardness recovery did not differ significantly among groups (*p* = 0.114). Because this variable was influenced by extreme values in specimens with small post-bleaching reductions, absolute ΔVHN was considered the more robust descriptive measure of remineralization response.

### 2.3. SEM–EDX Analysis

SEM–EDX analyses were performed to evaluate the surface morphological and elemental changes associated with the different remineralization protocols. SEM findings were evaluated descriptively, whereas EDX-derived Ca, P, and Ca/P values were subjected to quantitative and statistical analyses.

#### 2.3.1. SEM Surface Morphology

**Figure 1:**
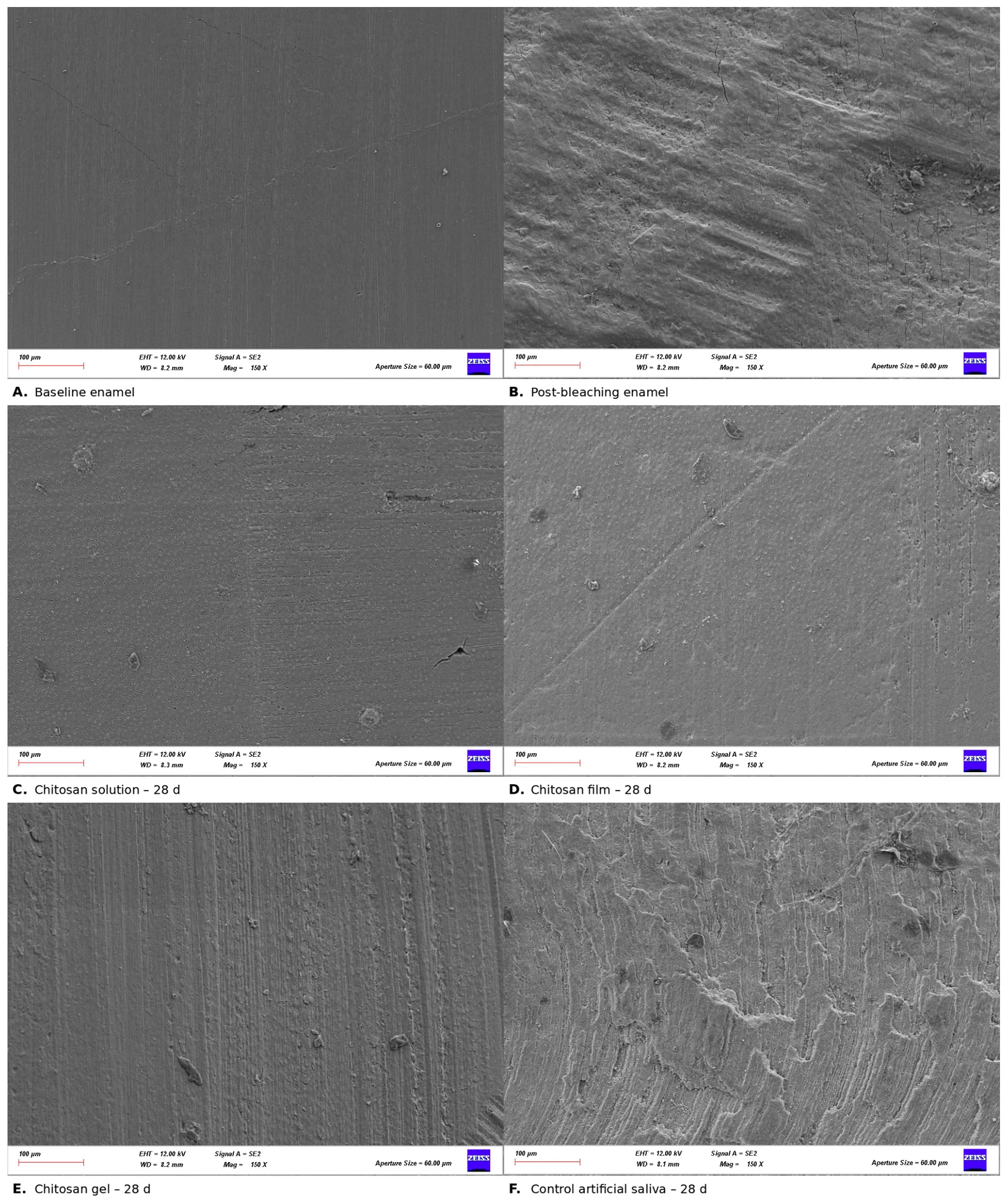
Representative scanning electron microscopy (SEM) micrographs of enamel surfaces at different experimental stages and after the 28-day remineralization protocols. (**A**) Baseline enamel; (**B**) post-bleaching enamel; (**C**) chitosan solution; (**D**) chitosan film; (**E**) chitosan gel; and (**F**) control artificial saliva after 28 days of remineralization. All panels are shown at 150× magnification with a 100 µm scale bar.

SEM examination showed marked morphological differences across the experimental stages. Baseline enamel exhibited a comparatively regular and compact surface, whereas post-bleaching enamel showed increased surface irregularity and disruption. Following the 28-day remineralization period, varying degrees of surface reorganization were observed among the experimental groups. The chitosan-containing groups showed morphological evidence of recovery of the altered surface, although the pattern differed among formulations. Because SEM provides primarily morphological information, these observations were interpreted descriptively and together with the quantitative Vickers microhardness and EDX findings (Figure 1).

#### 2.3.2. EDX Elemental Analysis

**Figure 2:**
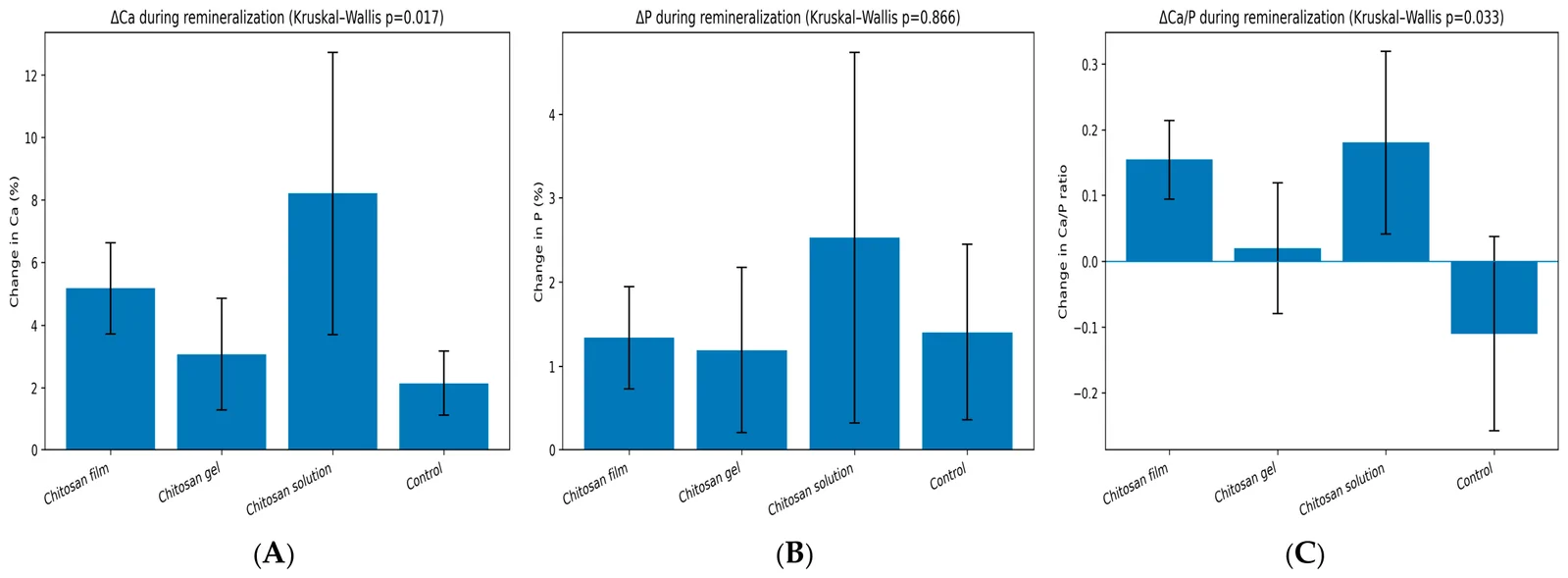
Changes in EDX-derived elemental parameters during the 28-day remineralization period. (**A**) calcium content (ΔCa), (**B**) phosphorus content (ΔP), and (**C**) Ca/P ratio (ΔCa/P) are presented as mean ± SD (*n* = 4 specimens per group). Δ values were calculated as T2 − T1. Between-group differences were significant for ΔCa (Kruskal–Wallis, *p* = 0.017) and ΔCa/P (*p* = 0.033), whereas ΔP did not differ significantly among groups (*p* = 0.866). No pairwise comparison remained statistically significant after Holm adjustment.

The EDX findings are presented in Table 2. Following bleaching, reductions in Ca and P contents were observed across the experimental groups. After 28 days of remineralization, both Ca and P contents increased in all groups. The increase in Ca content differed significantly among groups (Kruskal–Wallis, *p* = 0.017), with the greatest numerical Ca gain in the chitosan solution group, followed by film, gel, and control. Pairwise comparisons did not remain statistically significant after Holm adjustment (adjusted *p* > 0.05). Phosphorus increased in all groups, but ΔP did not differ significantly among groups (*p* = 0.866). Changes in the Ca/P ratio differed significantly among groups (*p* = 0.033); the greatest numerical increases occurred in the solution and film groups, the gel showed only a limited change, and the control showed a numerical reduction. Pairwise comparisons again did not remain significant after adjustment. These elemental changes broadly paralleled the microhardness findings and are presented graphically in Figure 2. Individual specimen-level EDX data are provided in Supplementary Table S2.

## 3. Discussion

The present study evaluated the influence of three physical forms of chitosan on the remineralization of bleached human enamel within a common olive leaf extract-containing artificial saliva system. Microhardness changed differently over time according to experimental group, and all chitosan-containing formulations produced greater ΔVHN than the control. The significant group × time interaction (*p* < 0.001; partial η^2^ = 0.274) indicates that the temporal pattern of enamel microhardness recovery was formulation-dependent rather than uniform across groups. The solution showed the greatest numerical gain and a significantly greater gain than the gel, whereas solution and film did not differ significantly. EDX findings were directionally consistent, with the greatest numerical Ca gain in the solution group and significant overall differences in ΔCa and ΔCa/P. Accordingly, the null hypothesis was rejected.

A marked reduction in enamel surface microhardness was observed following bleaching in all experimental groups. This finding is consistent with previous studies reporting that high-concentration hydrogen peroxide may adversely affect enamel mineral content and mechanical properties. Hydrogen peroxide and its reactive oxygen species can diffuse through the enamel structure and may alter the organic and inorganic components of the tissue, while the chemical characteristics of bleaching formulations may further contribute to mineral loss and increased enamel porosity [13]. The reduction observed after bleaching in the present study therefore confirms that the experimental protocol produced a measurable alteration in enamel microhardness and provided an appropriate substrate for evaluating subsequent remineralization.

A 28-day remineralization period was selected to evaluate the cumulative mineral recovery produced by the tested formulations rather than only their immediate surface effects. Similar 28-day protocols have been employed in in vitro enamel remineralization studies using microhardness and SEM–EDX analyses [14,15]. Furthermore, time-dependent SEM–EDX analysis has demonstrated progressive mineral recovery over 7, 14, 21, and 28 days, supporting 28 days as an appropriate endpoint for evaluating changes in enamel mineralization [16].

Following remineralization, surface microhardness increased significantly in all experimental groups. Importantly, the three chitosan-containing groups exhibited greater absolute microhardness gains than the control group, suggesting an additional contribution of chitosan beyond the remineralizing capacity of the artificial saliva itself. Chitosan is a bioactive cationic polymer with physicochemical properties that have supported its use in biomimetic mineralization strategies. Its functional groups allow interactions with dental hard tissues and bioactive components, providing a potential matrix for mineral deposition. A systematic review of in vitro studies concluded that chitosan-based systems show potential for enamel and dentin remineralization, although their efficacy varies according to formulation and experimental conditions [6]. In addition, Arnaud et al. demonstrated that chitosan treatment influenced enamel demineralization–remineralization behavior, with effects dependent on both concentration and exposure time [17]. The present findings extend these observations by indicating that the formulation in which chitosan is delivered may influence the magnitude of enamel microhardness recovery.

Recent evidence specifically investigating chitosan in bleached enamel further supports its protective potential. Ferraz et al. evaluated the incorporation of 2% chitosan into peroxide-based bleaching gels and reported favorable effects on enamel microhardness and surface properties [13]. Although chitosan was incorporated directly into the bleaching agent in that study, whereas it was applied during the post-bleaching remineralization phase in the present investigation, both approaches support the potential of chitosan to mitigate or promote recovery from bleaching-associated alterations in enamel properties.

Among the tested formulations, the chitosan solution demonstrated the greatest numerical microhardness gain and the highest final microhardness. This behavior may be related to the relatively low viscosity and homogeneous distribution of the solution, which may facilitate close interaction between chitosan, mineral ions, and the enamel surface. Following bleaching, increased enamel permeability and surface microporosity may provide sites into which a fluid formulation can readily penetrate. In addition, the greater mobility of calcium and phosphate ions in a solution compared with a more viscous matrix may facilitate ion diffusion toward the enamel surface and promote mineral deposition. The EDX findings support this interpretation, as the solution group exhibited the greatest numerical increase in Ca content and the greatest increase in the Ca/P ratio during remineralization.

The chitosan film also demonstrated substantial remineralization, with a microhardness gain that did not differ significantly from that of the solution. This finding may be related to the prolonged and intimate contact provided by the film at the enamel interface. The bioadhesive characteristics of chitosan-based materials may favor sustained surface interaction and provide a polymeric matrix capable of retaining bioactive components [6]. Consistent with the microhardness findings, the film group showed the second greatest numerical increases in Ca content and Ca/P ratio. Nevertheless, whether the film acted primarily through prolonged contact, mineral retention, or other interfacial mechanisms cannot be determined from the present data.

The gel formulation also enhanced enamel microhardness compared with the control, although its ΔVHN was significantly lower than that of the chitosan solution. The gel contained CMC as a viscosity-modifying excipient and exhibited a macroscopically more structured consistency than the solution. However, viscosity and rheological properties were not quantitatively measured. Therefore, any interpretation regarding reduced ionic mobility or diffusion within the gel should be regarded as a mechanistic hypothesis rather than an experimentally demonstrated rheological effect. The relatively limited change in Ca/P ratio in the gel group is compatible with, but does not prove, such a mechanism.

The control artificial saliva also promoted microhardness recovery after bleaching, indicating that the base formulation itself provided a remineralizing environment. This effect is biologically plausible because the artificial saliva contained calcium and phosphate sources capable of supporting mineral redeposition on altered enamel. Previous in vitro studies have likewise demonstrated enamel mineral recovery during exposure to remineralizing media containing available calcium and phosphate ions [14,16]. Nevertheless, final microhardness in the control group did not significantly exceed baseline values, and its absolute microhardness gain was significantly lower than that of all chitosan-containing groups. This suggests that chitosan provided an additional effect beyond that achieved by the artificial saliva alone.

The EDX findings complemented the microhardness data by demonstrating formulation-dependent changes in surface elemental composition. The numerical order of Ca gain (solution > film > gel > control) broadly paralleled the microhardness response, while increases in Ca/P ratio were most pronounced in the solution and film groups. These findings are compatible with enhanced mineral accumulation or retention at the enamel surface; however, EDX alone cannot establish the crystallographic nature of deposited mineral. Because only four specimens per group were analyzed and adjusted pairwise comparisons did not remain significant, the elemental findings should be regarded as supportive evidence of surface mineral recovery rather than direct proof of newly formed hydroxyapatite.

Olive leaf extract was incorporated as a common component of the artificial saliva in all experimental groups. Because an olive-leaf-extract-free artificial saliva group was not included, the present study cannot determine whether the extract independently enhanced enamel remineralization. Therefore, the observed between-group differences should primarily be interpreted as formulation-dependent effects associated with chitosan. The antioxidant and biological properties of olive leaf extract may remain relevant to the broader functionality of the artificial saliva platform, but its direct contribution to mineral recovery was not specifically tested in the present experimental design [18].

Several limitations should be considered. First, the in vitro design cannot reproduce salivary flow, acquired pellicle, pH fluctuations, dietary challenges, or mechanical forces. Second, specimens were continuously exposed to remineralization media for 28 days, whereas clinical use would likely involve intermittent exposure. Third, only four specimens per group underwent SEM–EDX analysis, so elemental findings should be considered complementary to the microhardness results. Fourth, the tested formulations differed not only in physical presentation but also in certain compositional features, including CMC in the gel and sodium bicarbonate in the solution; therefore, observed differences cannot be attributed exclusively to physical form. Fifth, quantitative viscosity/rheology, degree of deacetylation, and exact film thickness were not determined. Future studies should use compositionally standardized formulations, full physicochemical characterization, dynamic pH-cycling models, and clinically relevant application protocols.

Within these limitations, the present findings indicate that incorporating chitosan into an olive leaf extract-containing artificial saliva enhances surface microhardness recovery in bleached enamel, with the magnitude of recovery varying according to formulation. The solution and film showed particularly favorable outcomes, suggesting that formulation-dependent characteristics such as ion mobility and enamel contact may contribute to chitosan-mediated remineralization. Further studies are required to determine whether these effects are maintained under clinically relevant conditions.

## 4. Materials and Methods

### 4.1. Study Design

This in vitro experimental study was designed to compare the remineralization efficacy of three different physical forms of chitosan incorporated into an olive leaf extract-containing artificial saliva on bleached human enamel.

A total of 76 sound human anterior teeth extracted for periodontal or orthodontic reasons were collected from the Oral and Maxillofacial Surgery Clinic of Dokuz Eylül University Faculty of Dentistry. The study protocol was approved by the Non-Interventional Research Ethics Committee of Dokuz Eylül University (Approval No.: 2026/24-12). Teeth exhibiting caries, restorations, cracks, developmental defects, fluorosis, or any visible structural abnormalities were excluded from the study.

Following extraction, adherent soft tissue remnants and calculus deposits were carefully removed using hand instruments. The teeth were cleaned under running water and stored in distilled water to prevent dehydration until the experimental procedures. Prior to experimentation, each specimen was visually examined to confirm the integrity of the enamel surface.

The specimens were allocated according to the planned analyses. Sixty teeth were used for Vickers microhardness evaluation and randomly assigned to four experimental groups (*n* = 15 per group) using a computer-generated randomization sequence. Sixteen additional teeth were reserved for scanning electron microscopy (SEM) and energy-dispersive X-ray spectroscopy (EDX) analyses and distributed equally among the four groups (*n* = 4 per group).

The experimental groups were established as follows:

- Group 1: Chitosan film
- Group 2: Chitosan gel
- Group 3: Chitosan solution
- Group 4: Olive leaf extract-containing artificial saliva (control)

### 4.2. Preparation of Enamel Specimens

The roots of the extracted teeth were sectioned at the cemento-enamel junction using a low-speed diamond saw under continuous water cooling. The crown portions were subsequently embedded in self-curing acrylic resin blocks, leaving the labial enamel surfaces exposed for treatment and analysis.

To obtain standardized enamel surfaces, the exposed labial surfaces were sequentially polished under continuous water irrigation using 600-, 800-, and 1200-grit silicon carbide (SiC) abrasive papers. This procedure provided a flat and standardized enamel surface suitable for microhardness measurements and surface analyses.

A standardized enamel window measuring 4 × 4 mm was then delineated on the middle third of the labial surface of each specimen. The remaining enamel surfaces were coated with an acid-resistant nail varnish to prevent unwanted exposure to the bleaching and remineralization agents. Finally, all specimens were visually inspected to confirm the absence of surface defects before the experimental procedures.

### 4.3. Preparation of Olive Leaf Extract-Containing Artificial Saliva

An olive leaf extract-containing artificial saliva was prepared according to our previously developed biomimetic formulation, which was selected as the base remineralization medium for all experimental groups [19]. The formulation consisted of 0.33 g KH_2_PO_4_, 0.34 g Na_2_HPO_4_, 1.27 g KCl, 0.16 g NaSCN, 0.58 g NaCl, 0.17 g CaCl_2_, 0.16 g NH_4_Cl, 0.20 g urea, 0.03 g glucose, 0.002 g ascorbic acid, and 10 g sodium carboxymethyl cellulose (CMC) dissolved in 1000 mL of distilled water. Olive leaf extract (*Olea europaea* L.) was incorporated into the formulation as a natural phytochemical component to provide bioactive properties. The detailed composition and concentration of the olive leaf extract are not disclosed because the formulation is currently protected under a pending patent application.

The mixture was continuously stirred using a heated magnetic stirrer at 50 °C for 30 min to ensure complete dissolution and homogeneous distribution of all components. The final pH of the formulation was adjusted and stabilized at 6.80. Following preparation, the artificial saliva was sterilized using ultraviolet (UV) irradiation for 10 min under aseptic conditions prior to experimental use.

The prepared artificial saliva served as the remineralization medium for all experimental groups and constituted the sole treatment in the control group.

### 4.4. Preparation of Chitosan Formulations

To evaluate the influence of chitosan physical form on enamel remineralization, three formulations—film, gel, and solution—were prepared or selected for experimental use. A nominal chitosan concentration of 2% (*w*/*v*) was used for the gel and solution formulations based on previous dental applications of 2% chitosan and on preliminary formulation feasibility within the artificial saliva system [13]. The same nominal concentration was maintained in gel and solution formulations to permit comparison of different physical presentations. The formulations were not derived from a formal response-surface optimization design; rather, they were selected as comparative experimental formulations based on literature and practical formulation feasibility (Table 3).

#### 4.4.1. Chitosan Film

A commercially available medical-grade chitosan film dressing (MaxioCel, Axio Biosolutions Pvt. Ltd., Ahmedabad, India), composed of 100% Grade A chitosan, was used as the film formulation. The molecular weight of the chitosan has been reported to be approximately 224.7 ± 8.4 kDa; the bibliographic source for this value will be verified before final submission. The degree of deacetylation and exact film thickness were not available in the manufacturer information accessible to the authors and were not independently determined in the present study. Before application, the film was trimmed to fit the standardized 4 × 4 mm enamel window and carefully adapted to the labial enamel surface. During the remineralization period, specimens were immersed in the olive leaf extract-containing artificial saliva while maintaining film–enamel contact, and the film was replaced daily.

#### 4.4.2. Chitosan Gel

A 2% (*w*/*v*) chitosan gel was prepared using chitosan powder (Sepe Natural Organic Products, Izmir, Türkiye). Two grams of chitosan were dispersed in the olive leaf extract-containing artificial saliva in the presence of 0.5 mL acetic acid and 1.5 mL distilled water to facilitate dissolution. Subsequently, 4 g of sodium carboxymethyl cellulose was incorporated as a gel-forming and viscosity-modifying excipient. The final volume was adjusted to 100 mL, corresponding to 2% (*w*/*v*) chitosan and 4% (*w*/*v*) CMC. The preparation was mixed until a macroscopically homogeneous gel was obtained, and the final pH was adjusted to 6.15. Quantitative viscosity and rheological measurements were not performed.

#### 4.4.3. Chitosan Solution

A 2% (*w*/*v*) chitosan solution was prepared using 2 g of chitosan powder (Sepe Natural Organic Products, Türkiye) in a final volume of 100 mL of olive leaf extract-containing artificial saliva. To facilitate dissolution, 0.5 mL acetic acid and 1.5 mL distilled water were added. Subsequently, 600 μL sodium bicarbonate was added to partially neutralize the acidic environment. The final pH was 6.15. The solution was continuously mixed until macroscopically homogeneous and was freshly prepared prior to use. Quantitative viscosity and rheological measurements were not performed.

### 4.5. Bleaching Procedure

Following enamel specimen preparation, all specimens were subjected to an in-office bleaching procedure using a 35% hydrogen peroxide gel (Whiteness HP, FGM Produtos Odontológicos, Joinville, SC, Brazil). A 35% hydrogen peroxide concentration was selected to reproduce a clinically relevant in-office bleaching challenge and to generate measurable post-bleaching alterations in enamel surface properties, as reported in previous laboratory studies and reviews of high-concentration peroxide bleaching [2,13]. The bleaching treatment was performed according to the manufacturer’s recommendations.

The bleaching gel was applied to the standardized enamel windows in three consecutive applications of 15 min each, resulting in a total exposure time of 45 min. At the end of each 15-min application, the bleaching gel was thoroughly removed by rinsing with distilled water, and a freshly mixed bleaching gel was applied for the subsequent application.

At the completion of the bleaching procedure, all specimens were thoroughly rinsed with distilled water and gently air-dried. Following bleaching, the specimens were immediately assigned to their respective remineralization protocols according to the experimental group allocation.

### 4.6. Remineralization Protocol

Following the bleaching procedure, the specimens were randomly allocated to the four experimental groups and subjected to remineralization treatment for 28 days under controlled laboratory conditions.

In the chitosan film group, a commercially available chitosan film was adapted onto the enamel surface covering the standardized treatment window. During the 28-day remineralization period, the specimens were immersed in the olive leaf extract-containing artificial saliva while maintaining continuous contact between the enamel surface and the chitosan film, which was replaced daily. In the chitosan gel group, the specimens were directly immersed in the prepared 2% chitosan gel. Specimens in the chitosan gel and chitosan solution groups were immersed directly in the corresponding 2% formulations throughout the remineralization period. Specimens in the control group were immersed exclusively in the olive leaf extract-containing artificial saliva without any chitosan supplementation.

All specimens were maintained at 37 °C in an incubator to simulate intraoral conditions. The artificial saliva was renewed daily in all groups to maintain the physicochemical stability of the storage medium throughout the experimental period. After completion of the 28-day remineralization period, the specimens were rinsed with distilled water and prepared for Vickers microhardness, scanning electron microscopy (SEM), and energy-dispersive X-ray spectroscopy (EDX) analyses (Table 4).

### 4.7. Vickers Microhardness Analysis

Surface microhardness measurements were performed using a Vickers microhardness tester (HMV-700 Micro Hardness Tester, Shimadzu Corp., Kyoto, Japan). Fifteen independent specimens were analyzed per group (*n* = 15/group). Measurements were obtained at T0 (baseline), T1 (immediately after bleaching), and T2 (after 28 days of remineralization). Three indentations were created on each specimen at each time point using a 200 gf load for 10 s. The indentations were placed at standardized distances to avoid overlapping stress fields and edge effects. The mean of the three technical measurements was used as the specimen-level VHN for statistical analysis.

### 4.8. Scanning Electron Microscopy (SEM) Analysis

Surface morphological alterations were evaluated using scanning electron microscopy (SEM) (GeminiSEM 560, Carl Zeiss AG, Oberkochen, Germany). Four independent specimens were examined per experimental group (*n* = 4/group). Specimens were prepared according to the instrument protocol, mounted on aluminum stubs, and examined at an accelerating voltage of 12 kV under different magnifications. Representative micrographs were selected to illustrate surface morphology. SEM findings were evaluated descriptively and were not treated as independent quantitative replicates.

### 4.9. Energy-Dispersive X-Ray Spectroscopy (EDX) Analysis

Elemental analysis was performed using an energy-dispersive X-ray spectroscopy (EDX) system coupled to the SEM. Four independent specimens were analyzed per experimental group (*n* = 4/group). Calcium (Ca) and phosphorus (P) contents were determined and Ca/P ratios were calculated. Two EDX measurements were obtained from each specimen under standardized operating conditions, and their mean was used as the specimen-level value for statistical analysis.

### 4.10. Statistical Analysis

Statistical analyses were performed using IBM SPSS Statistics for Windows, version 30.0 (IBM Corp., Armonk, NY, USA). For Vickers microhardness, the mean of three indentations from each specimen was used as the statistical unit. Data distribution and homogeneity of variances were assessed using the Shapiro–Wilk and Levene tests. Changes in microhardness were evaluated using mixed-design repeated-measures ANOVA, with time (T0, T1, T2) as the within-subject factor and experimental formulation (film, gel, solution, control) as the categorical between-subject factor. Thus, the group × time interaction was directly tested. Sphericity was assessed using Mauchly’s test, with Greenhouse–Geisser correction when required. Effect sizes were expressed as partial η^2^, and pairwise comparisons used Bonferroni adjustment. ΔVHN (T2 − T1) was compared among groups using Welch’s ANOVA, followed by adjusted pairwise comparisons. Percentage microhardness recovery was analyzed using the Kruskal–Wallis test because of its non-normal distribution. SEM micrographs were evaluated descriptively. For EDX, the mean of two measurements from each specimen was used as the statistical unit; ΔCa, ΔP, and ΔCa/P were compared among groups using Kruskal–Wallis tests, followed where appropriate by exact Mann–Whitney U tests with Holm adjustment. Because the study was designed as a comparative categorical four-group experiment rather than a factorial optimization study, response-surface modeling was not considered statistically appropriate.

SEM micrographs were evaluated descriptively for surface morphology and mineral deposition patterns. For EDX analysis, the mean of two measurements obtained from each specimen was used as the statistical unit. Changes in Ca and P contents and the Ca/P ratio during remineralization (T2 − T1) were compared among groups using the Kruskal–Wallis test. Significant overall differences were further evaluated using exact Mann–Whitney U tests with Holm adjustment. Within-group changes across T0, T1, and T2 were explored using the Friedman test. All tests were two-sided, and statistical significance was set at *p* < 0.05.

## 5. Conclusions

Within the limitations of this in vitro study, chitosan-containing formulations enhanced enamel surface microhardness recovery compared with the common olive leaf extract-containing artificial saliva platform alone. The chitosan solution showed the greatest numerical microhardness gain, while the film produced a comparable response. SEM–EDX findings provided complementary evidence of surface mineral recovery, with the solution and film showing the most favorable numerical changes in Ca content and Ca/P ratio. Because olive leaf extract was present in all groups, its independent contribution to remineralization cannot be determined from the present design. Overall, formulation-dependent characteristics appear to influence the remineralization response and warrant further study under clinically relevant conditions.

## Figures and Tables

**Table 1:** Mean (±SD) Vickers surface microhardness (VHN) values of the experimental groups at baseline (T0), after bleaching (T1), after remineralization (T2), and the corresponding microhardness gain (ΔVHN).

Experimental Group	T0, Mean ± SD	T1, Mean ± SD	T2, Mean ± SD	ΔVHN, Mean ± SD
Chitosan film	361.52 ± 96.34	225.93 ± 109.83	437.49 ± 56.31	211.56 ± 95.38
Chitosan gel	359.59 ± 38.85	216.04 ± 60.28	404.96 ± 32.03	188.92 ± 56.75
Chitosan solution	353.39 ± 63.85	200.15 ± 47.40	452.07 ± 43.90	251.92 ± 46.57
Control artificial saliva	383.40 ± 51.68	291.69 ± 69.60	398.71 ± 46.22	107.02 ± 54.09
*p* value	0.627	0.003 ^a^	0.005	<0.001 ^a^

Values are presented as mean ± standard deviation. T0: baseline; T1: immediately after bleaching; T2: following the 28-day remineralization period; ΔVHN: T2 − T1. ^a^ Welch’s ANOVA.

**Table 2:** EDX-derived calcium (Ca), phosphorus (P), and Ca/P ratio values after bleaching (T1), after remineralization (T2), and their changes during the remineralization period.

Experimental Group	Ca T1	Ca T2	ΔCa	P T1	P T2	ΔP	Ca/P T1	Ca/P T2	ΔCa/P
Chitosan film	26.85 ± 3.67	32.03 ± 3.90	5.18 ± 1.45	11.31 ± 1.49	12.65 ± 1.32	1.34 ± 0.61	2.375 ± 0.098	2.529 ± 0.080	0.154 ± 0.060
Chitosan gel	28.86 ± 1.73	31.93 ± 1.76	3.06 ± 1.79	12.63 ± 1.08	13.81 ± 0.59	1.19 ± 0.99	2.291 ± 0.082	2.310 ± 0.036	0.020 ± 0.099
Chitosan solution	25.25 ± 4.26	33.46 ± 2.93	8.21 ± 4.51	11.61 ± 2.50	14.14 ± 1.62	2.53 ± 2.21	2.193 ± 0.160	2.374 ± 0.086	0.180 ± 0.139
Control artificial saliva	25.10 ± 2.09	27.24 ± 2.04	2.14 ± 1.03	10.41 ± 0.98	11.81 ± 0.62	1.40 ± 1.05	2.415 ± 0.130	2.304 ± 0.074	−0.111 ± 0.148
*p* value	—	—	0.017	—	—	0.866	—	—	0.033

Values are presented as mean ± standard deviation (*n* = 4 independent specimens per group). For each specimen, the mean of two EDX measurements was used as the statistical unit. T1: immediately after bleaching; T2: following the 28-day remineralization period; ΔCa, ΔP, and ΔCa/P: T2 − T1. Between-group differences in changes were evaluated using the Kruskal–Wallis test. Significant overall differences were further examined using exact Mann–Whitney U tests with Holm adjustment; no pairwise comparison remained statistically significant after adjustment (adjusted *p* > 0.05).

**Table 3:** Physicochemical and compositional characteristics of the chitosan formulations used in the study.

Characteristic	Chitosan Film	Chitosan Gel	Chitosan Solution
Source	MaxioCel, Axio Biosolutions	Sepe Natural	Sepe Natural
Physical form	Medical-grade film	Gel	Solution
Chitosan concentration	Commercial 100% Grade A chitosan film	2% (*w*/*v*)	2% (*w*/*v*)
CMC	Not applicable	4% (*w*/*v*)	None
Acetic acid	Not applicable	0.5 mL/100 mL	0.5 mL/100 mL
Distilled water	—	1.5 mL/100 mL	1.5 mL/100 mL
Sodium bicarbonate	—	—	600 μL/100 mL
Final pH	Not determined	6.15	6.15
Molecular weight	~224.7 ± 8.4 kDa *	Not provided	Not provided
Degree of deacetylation	Not provided	Not provided	Not provided
Thickness	Not independently measured	N/A	N/A
Viscosity/rheology	N/A	Not quantitatively measured	Not quantitatively measured

* Reported value for the commercial medical-grade chitosan; the bibliographic source should be verified before final submission.

**Table 4:** Experimental design, factors, levels, and outcome measures.

Design Component	Specification
Study design	In vitro four-group comparative study
Total teeth	76
Vickers specimens	60; *n* = 15/group
SEM–EDX specimens	16; *n* = 4/group
Between-subject factor	Experimental formulation
Factor levels	Film; gel; solution; control artificial saliva
Within-subject factor (Vickers)	Time
Time levels	T0 baseline; T1 post-bleaching; T2 post-remineralization
Primary response	Vickers surface microhardness
Derived Vickers response	ΔVHN = T2 − T1
SEM response	Surface morphology (descriptive)
EDX responses	Ca, P, Ca/P
Derived EDX responses	ΔCa, ΔP, ΔCa/P
Remineralization period	28 days
Incubation temperature	37 °C

## Data Availability

The data supporting the findings of this study are available from the corresponding author, upon reasonable request.

## Supplementary Materials

The following supporting information can be downloaded at https://www.mdpi.com/article/10.3390/molecules31203601/s1, Table S1: Individual specimen-level Vickers microhardness data; Table S2: Individual specimen-level EDX elemental data.

## Abbreviations

The following abbreviations are used in this manuscript:

Ca	Calcium
Ca/P	Calcium-to-phosphorus ratio
CMC	Carboxymethyl cellulose
EDX	Energy-dispersive X-ray spectroscopy
P	Phosphorus
SEM	Scanning electron microscopy
SEM–EDX	Scanning electron microscopy coupled with energy-dispersive X-ray spectroscopy
SiC	Silicon carbide
VHN	Vickers hardness number
ΔVHN	Change in Vickers hardness number (T2 − T1)
T0	Baseline measurement
T1	Post-bleaching measurement
T2	Post-remineralization measurement
UV	Ultraviolet
